# Influence of *MUC5B* gene on antisynthetase syndrome

**DOI:** 10.1038/s41598-020-58400-0

**Published:** 2020-01-29

**Authors:** Raquel López-Mejías, Sara Remuzgo-Martínez, Fernanda Genre, Verónica Pulito-Cueto, Sonia M. Fernández Rozas, Javier Llorca, David Iturbe Fernández, Víctor M. Mora Cuesta, Norberto Ortego-Centeno, Nair Pérez Gómez, Antonio Mera-Varela, Julia Martínez-Barrio, Francisco Javier López-Longo, Verónica Mijares, Leticia Lera-Gómez, María Piedad Usetti, Rosalía Laporta, Virginia Pérez, Alicia De Pablo Gafas, María Aránzazu Alfranca González, Jaime Calvo-Alén, Fredeswinda Romero-Bueno, Olga Sanchez-Pernaute, Laura Nuno, Gema Bonilla, Alejandro Balsa, Fernanda Hernández-González, Ignacio Grafia, Sergio Prieto-González, Javier Narvaez, Ernesto Trallero-Araguas, Albert Selva-O’Callaghan, Oreste Gualillo, Santos Castañeda, Lorenzo Cavagna, José M. Cifrian, Miguel A. González-Gay

**Affiliations:** 1 Epidemiology, Genetics and Atherosclerosis Research Group on Systemic Inflammatory Diseases, Hospital Universitario Marqués de Valdecilla, IDIVAL, Santander, Spain; 2 Department of Epidemiology and Computational Biology, School of Medicine, University of Cantabria, and CIBER Epidemiología y Salud Pública (CIBERESP), IDIVAL, Santander, Spain; 3 Systemic Autoimmune Diseases Unit, Hospital Universitario San Cecilio, Granada, Spain; 4 Division of Rheumatology, Hospital Clínico Universitario de Santiago, Santiago de Compostela, Spain; 5 Department of Rheumatology, Hospital General Universitario Gregorio Marañón, Madrid, Spain; 6 Pneumology Department, Hospital Universitario Puerta de Hierro, Madrid, Spain; 7 Lung Transplant Unit, Division of Pulmonology, Hospital Universitario 12 de Octubre, Madrid, Spain; 8 Department of Immunology, Hospital Universitario de la Princesa, IIS-Princesa, Madrid, Spain; 9 Rheumatology Division, Hospital Universitario Araba, Universidad del País Vasco, Vitoria, Spain; 10 Rheumatology Department, Bone and Joint Research Unit, Hospital Universitario Fundación Jiménez Díaz, IIS Fundación Jiménez Díaz, Universidad Autónoma de Madrid, Madrid, Spain; 11 Rheumatology Department, Hospital Universitario La Paz, Madrid, Spain; 12 Respiratory Department, Hospital Clínico de Barcelona, Barcelona, Spain; 13 Department of Autoimmune Diseases, Hospital Clínico de Barcelona, Universidad de Barcelona, Barcelona, Spain; 14 Rheumatology Department, Hospital Universitario de Bellvitge, Barcelona, Spain; 15 Department of Systemic Autoimmune Diseases, Hospital Universitario Valle de Hebron, Universidad Autónoma de Barcelona, Barcelona, Spain; 16 Servizo Galego de Saude and Instituto de Investigación Sanitaria-Hospital Clínico Universitario de Santiago, Santiago de Compostela, Spain; 17 Rheumatology Department, Hospital Universitario de la Princesa, IIS-Princesa, Madrid, Spain; 18 Division of Rheumatology, University and IRCCS Policlinico S. Matteo Foundation of Pavia and ERN ReCONNET, Pavia, Italy; 19 School of Medicine, Universidad de Cantabria, Santander, Spain; 20 Cardiovascular Pathophysiology and Genomics Research Unit, School of Physiology, Faculty of Health Sciences, University of the Witwatersrand, Johannesburg, South Africa

**Keywords:** Rheumatology, Rheumatic diseases

## Abstract

*MUC5B* rs35705950 (G/T) is strongly associated with idiopathic pulmonary fibrosis (IPF) and also contributes to the risk of interstitial lung disease (ILD) in rheumatoid arthritis (RA-ILD) and chronic hypersensitivity pneumonitis (CHP). Due to this, we evaluated the implication of *MUC5B* rs35705950 in antisynthetase syndrome (ASSD), a pathology characterised by a high ILD incidence. 160 patients with ASSD (142 with ILD associated with ASSD [ASSD-ILD+]), 232 with ILD unrelated to ASSD (comprising 161 IPF, 27 RA-ILD and 44 CHP) and 534 healthy controls were genotyped. *MUC5B* rs35705950 frequency did not significantly differ between ASSD-ILD+ patients and healthy controls nor when ASSD patients were stratified according to the presence/absence of anti Jo-1 antibodies or ILD. No significant differences in *MUC5B* rs35705950 were also observed in ASSD-ILD+ patients with a usual interstitial pneumonia (UIP) pattern when compared to those with a non-UIP pattern. However, a statistically significant decrease of *MUC5B* rs35705950 GT, TT and T frequencies in ASSD-ILD+ patients compared to patients with ILD unrelated to ASSD was observed. In summary, our study does not support a role of *MUC5B* rs35705950 in ASSD. It also indicates that there are genetic differences between ILD associated with and that unrelated to ASSD.

## Introduction

Mucins are components of mucus secretions with a crucial implication in the host defence against bacterial and fungal infections^1^. Mucin 5B is a gel-forming mucin and a major constituent of mucus in the respiratory tract^2^ that is encoded by *MUC5B*^3^. A common gain-of-function variant located in the promoter of this gene, *MUC5B* rs35705950, is described as the strongest genetic risk factor for the development of idiopathic pulmonary fibrosis (IPF)^4^, the most common and pernicious form of interstitial lung disease (ILD)^5^. A recent study performed by Juge *et al*. has revealed that *MUC5B* rs35705950 also significantly contributes to the risk of ILD amongst patients with rheumatoid arthritis (RA-ILD)^6^. In addition, *MUC5B* rs35705950 has been previously associated with chronic hypersensitivity pneumonitis (CHP), a relevant finding reported by Ley *et al*.^7^.

Antisynthetase syndrome (ASSD) is a connective tissue disease characterized by the typical clinical triad of arthritis, myositis and ILD^8–13^. Amongst these clinical features, ILD has been identified as the most frequent^14^ and severe^15^ manifestation of ASSD, with an incidence of approximately 80–90%^16,17^. The type and severity of ILD defines the long-term outcome of ASSD^10^, being the major determinant of morbidity and mortality in patients affected with this condition^10,17–22^. The pathophysiology of ASSD is not entirely understood, although some pieces of evidence support the hypothesis that both genetic and environmental factors may play a relevant role^23^.

Taking all these considerations into account, we aimed to determine whether the *MUC5B* rs35705950 promoter polymorphism, associated with IPF^4^, RA-ILD^6^ and CH^7^, was also implicated in the pathogenesis of ASSD. For this purpose, we took advantage of data from one of the largest cohorts of patients with ASSD.

## Patients and Methods

### Patients and study protocol

A total of 160 unrelated Spanish patients of European ancestry (self-reported) diagnosed with ASSD were enrolled in this study. Centres involved in the recruitment of these patients included Hospital Universitario Marqués de Valdecilla (Santander), Hospital Universitario San Cecilio (Granada), Hospital Clínico Universitario de Santiago (Santiago de Compostela), Hospital Universitario Araba (Vitoria), Hospital Clínico de Barcelona, Hospital Universitario de Bellvitge and Hospital Universitario Valle de Hebrón (Barcelona), and Hospital General Universitario Gregorio Marañón, Hospital Universitario Fundación Jiménez Díaz, Hospital Universitario La Paz and Hospital Universitario de la Princesa (Madrid). Patients were recruited if they had an antisynthetase antibody testing positive in at least two determinations along with one or more findings of the typical clinical triad (arthritis, myositis and/or ILD). Briefly, arthritis occurrence and its presentation pattern were clinically assessed by the referent physician; myositis was defined in case of muscle enzyme elevation (creatinine phosphokinase and/or aldolase) and the presence of typical electromyography alterations and/or compatible muscle biopsy findings and/or compatible muscle magnetic resonance; ILD was defined instrumentally by a restrictive pulmonary function test pattern [forced vital capacity (FVC) ≤ 80%, forced expiratory volume in one second (FEV1)/FVC ≥ 70%, decreased/normal FEV1, and/or diffusing capacity of the lung for carbon monoxide (DLCO) reduction >20%] and/or by the identification of alveolitis/fibrosis signs at high-resolution computed tomography of the lungs^8,9,12,24,25^. According to these criteria, 142 (88.8%) patients developed ILD associated with ASSD (ASSD-ILD+). Among them, 70% showed a non-specific interstitial pneumonia (NSIP) pattern in the high-resolution computed tomography (HRCT) images of the chest, while 19% of the patients showed a usual interstitial pneumonia (UIP) pattern. The remaining 11% of the ASSD-ILD+ patients presented other patterns. The occurrence of accompanying features, including fever, Raynaud´s phenomenon and mechanic’s hands, were also assessed as previously described^11,18^. Fever was considered in case of a body temperature ≥38 °C for more than 10 days without evidence of any other reason. Raynaud’s phenomenon was determined as the occurrence of a transient finger ischemia after cold exposure. Mechanic’s hands were defined as the occurrence of a thickened, hyperkeratotic, and fissured aspect of the radial sides of the fingers of the hands, in absence of other causes^12,19^. A detailed description of the main demographic and clinical information of the patients with ASSD enrolled in this study is displayed in Table 1.

**Table 1:** Main demographic and clinical information of the 160 patients diagnosed with ASSD included in this study.

	[% (n/N)]
Median age in years at disease onset [IQR]	48.0 [37.0–58.7]
Median follow-up in months [IQR]	81.5 [45.0–170.5]
Percentage of females	69.7
Antisynthetase antibody	100.0 (160/160)
Anti Jo-1 positive	65.0 (104/160)
Arthritis	62.5 (100/160)
Myositis	69.4 (111/160)
ILD	88.8 (142/160)
Fever	34.4 (53/154)
Raynaud’s phenomenon	37.7 (58/154)
Mechanic’s hands	56.5 (87/154)

ASSD: antisynthetase syndrome; IQR: interquartile range; ILD: interstitial lung disease.

In addition, a set of 232 ethnically matched (self-reported) patients diagnosed with ILD unrelated to ASSD was also included in this work. Centres involved in the recruitment of these patients included Hospital Universitario Marqués de Valdecilla (Santander), and Hospital Universitario Puerta de Hierro and Hospital Universitario 12 de Octubre (Madrid). Amongst these patients, 161 (69.4%) exhibited IPF, according to the classification criteria for the disease^26^, whereas 27 (11.6%) experienced RA-ILD and 44 (19.0%) exhibited CHP.

Moreover, a total of 534 ethnically (self-reported) matched unaffected control subjects, without history of any autoimmune or pulmonary disease, constituted by blood donors from Hospital Universitario Marqués de Valdecilla (Santander) and National DNA Bank Repository (Salamanca), was also enrolled in the study.

All patients and healthy controls signed an informed written consent before being included in the study, according to the declaration of Helsinki. The procedures followed were in accordance with the ethical standards of the approved guidelines and regulations, according to the Declaration of Helsinki. All experimental protocols were approved by the Ethics Committees of clinical research of Cantabria for Hospital Universitario Marqués de Valdecilla in Santander, of Andalucía for Hospital Universitario San Cecilio in Granada, of Galicia for Hospital Clínico Universitario de Santiago in Santiago de Compostela, of País Vasco for Hospital Universitario Araba in Vitoria, of Cataluña for Hospital Clínico de Barcelona, Hospital Universitario de Bellvitge and Hospital Universitario Valle de Hebrón in Barcelona, and of Madrid for Hospital General Universitario Gregorio Marañón, Hospital Universitario Fundación Jiménez Díaz, Hospital Universitario La Paz, Hospital Universitario de la Princesa, Hospital Universitario Puerta de Hierro and Hospital Universitario 12 de Octubre in Madrid.

### Single nucleotide polymorphism selection and genotyping

The common gain-of-function genetic variant *MUC5B* rs35705950 (G/T), described as the strongest risk factor for IPF^4^ that also contributes to the risk of RA-ILD^6^, was selected in this study.

Genomic deoxyribonucleic acid from patients with ASSD, patients with ILD unrelated to ASSD and healthy controls was extracted from peripheral blood using standard procedures. All patients and healthy controls were genotyped for the *MUC5B* polymorphism mentioned above using a predesigned TaqMan 5′ single-nucleotide polymorphism genotyping assay (C_1582254_20) in a QuantStudio^TM^ 7 Flex Real-Time polymerase chain reaction system, according to the conditions recommended by the manufacturer (Applied Biosystems, Foster City, CA, USA).

Negative controls and duplicate samples were included to check the accuracy of the genotyping.

### Statistical analysis

Genotype data were checked for deviation from Hardy-Weinberg equilibrium (HWE) by chi-square test.

Both genotype and allele frequencies of *MUC5B* rs35705950 were calculated and compared between ASSD-ILD+ patients and healthy controls, patients with ASSD stratified according to specific clinical features of the disease (presence/absence of anti Jo-1 antibodies or ILD), ASSD-ILD+ patients stratified according to the presence of an UIP and non-UIP HRCT pattern, as well as between ASSD-ILD+ patients and those with ILD unrelated to ASSD.

To test for association, 3 × 2 and 2 × 2 contingency tables as well as chi-square test and/or Fisher´s exact test, when appropriate, were used. Strength of associations were estimated using odds ratios and 95% confidence intervals. P-values lower than 0.05 were considered as statistically significant.

All analyses were performed with STATA statistical software 12/SE (Stata Corp., College Station, TX, USA).

## Results

The genotyping success rate was greater than 99%.

No evidence of departure from HWE was observed in healthy controls at the 5% significance level. Genotype and allele frequencies of *MUC5B* rs35705950 in healthy controls were similar to those reported for populations of European origin in the 1000 Genomes Project (http://www.internationalgenome.org/).

### Differences in genotype and allele frequencies of *MUC5B* rs35705950 between ASSD-ILD+patients and healthy controls

Firstly, we compared genotype and allele frequencies of *MUC5B* rs35705950 between ASSD-ILD+ patients and healthy controls.

As shown in Table 2, no statistically significant differences in the genotype frequencies of *MUC5B* rs35705950 were disclosed when ASSD-ILD+ patients were compared to healthy controls. Likewise, allele frequencies of *MUC5B* rs35705950 did not significantly differ between ASSD-ILD+ patients and healthy controls (Table 2).

**Table 2:** Genotype and allele frequencies of *MUC5B* rs35705950 in ASSD-ILD + patients and healthy controls.

	ASSD-ILD + % (n)	Healthy controls % (n)	*p*	OR [95% CI]
***MUC5B*** **rs35705950**				
**Genotypes**				
GG	79.6 (113)	77.2 (412)	—	ref.
GT	19.7 (28)	21.2 (113)	0.67	0.90 [0.55–1.46]
TT	0.7 (1)	1.7 (9)	0.38	0.41 [0.01–2.98]
**Alleles**				
G	89.4 (254)	87.7 (937)	—	ref.
T	10.6 (30)	12.3 (131)	0.43	0.84 [0.54–1.30]

ASSD: antisynthetase syndrome; ILD: interstitial lung disease; OR: odds ratio; CI: confidence interval.

### Differences in genotype and allele frequencies of *MUC5B* rs35705950 between patients with ASSD stratified according to specific clinical features of the disease

In a further step, we analysed potential differences in the genotype and allele frequencies of *MUC5B* rs35705950 between patients with ASSD stratified according to the presence/absence of anti Jo-1 antibodies or ILD.

No genotype or allele differences in *MUC5B* rs35705950 were observed when patients with ASSD were stratified according to the presence/absence of anti Jo-1 antibodies (Table 3). It was also the case when patients with ASSD who developed ILD were compared to those who did not exhibit this pulmonary complication (Table 3).

**Table 3:** Genotype and allele frequencies of *MUC5B* rs35705950 in patients with ASSD stratified according to the presence/absence of anti-Jo-1 antibodies or ILD.

	ASSD		*p*	OR [95% CI]	ASSD		*p*	OR [95% CI]
	anti Jo-1 + % (n)	anti Jo-1- % (n)			ILD + % (n)	ILD- % (n)		
***MUC5B*** **rs35705950**								
**Genotypes**								
GG	83.7 (87)	73.2 (41)	—	Ref.	79.6 (113)	83.3 (15)	—	ref.
GT	15.4 (16)	25.0 (14)	0.13	0.54 [0.22–1.32]	19.7 (28)	11.1 (2)	0.42	1.85 [0.39–17.63]
TT	1.0 (1)	1.8 (1)	0.59	0.47 [0.006–37.88]	0.7 (1)	5.6 (1)	0.10	0.13 [0.002–11.11]
**Alleles**								
G	91.3 (190)	85.7 (96)	—	Ref.	89.4 (254)	88.9 (32)	—	ref.
T	8.7 (18)	14.3 (16)	0.12	0.57 [0.26–1.25]	10.6 (30)	11.1 (4)	0.92	0.94 [0.30–3.93]

ASSD: antisynthetase syndrome; ILD: interstitial lung disease; OR: odds ratio; CI: confidence interval.

### Differences in genotype and allele frequencies of *MUC5B* rs35705950 between ASSD-ILD+ patients stratified according to the presence of an UIP and non-UIP HRCT pattern”

Moreover, we also evaluated potential differences in the genotype and allele frequencies of *MUC5B* rs35705950 between ASSD-ILD+ patients stratified according to the presence of an UIP and non-UIP HRCT pattern.

No genotype or allele differences in *MUC5B* rs35705950 were observed in ASSD-ILD+ patients with an UIP pattern when compared to those with a non-UIP pattern (Table 4).

**Table 4:** Genotype and allele frequencies of *MUC5B* rs35705950 in ASSD-ILD + patients stratified according to the presence of an UIP and non-UIP pattern.

	UIP % (n)	Non-UIP % (n)	*p*	OR [95% CI]
***MUC5B*** **rs35705950**				
**Genotypes**				
GG	70.4 (19)	81.1 (86)	—	ref.
GT	29.6 (8)	17.9 (19)	0.19	1.91 [0.62–5.41]
TT	0.0 (0)	1.0 (1)	—	—
**Alleles**				
G	85.2 (46)	90.1 (191)	—	ref.
T	14.8 (8)	9.9 (21)	0.30	1.58 [0.57–4.01]

ASSD: antisynthetase syndrome; ILD: interstitial lung disease; UIP: usual interstitial pneumonia; OR: odds ratio; CI: confidence interval.

### Differences in genotype and allele frequencies of *MUC5B*rs35705950 between ASSD-ILD+ patients and patients with ILD unrelated to ASSD

We also examined whether genotype or allele frequencies of *MUC5B* rs35705950 differed between patients with ASSD-ILD+ and those with ILD unrelated to ASSD.

Interestingly, we disclosed a statistically significant decrease of *MUC5B* rs35705950 GT, TT and T frequencies in patients with ASSD-ILD + compared to the whole cohort of patients with ILD unrelated to ASSD (GT: 19.7% *versus* 53.4%, p < 0.0001; TT: 0.7% *versus* 9.1%, p < 0.0001; T: 10.6% *versus* 35.8%, p < 0.0001) (Supplementary Table 1). This significant decrease was also observed when patients with ASSD-ILD + were compared to those with IPF (GT: 19.7% *versus* 58.8%, p < 0.0001; TT: 0.7% *versus* 6.9%, p < 0.0001; T: 10.6% *versus* 36.0%, p < 0.0001), RA-ILD (GT: 19.7% *versus* 40.7%, p = 0.006; TT: 0.7% *versus* 11.1%, p = 0.0001; T: 10.6% *versus* 31.5% p < 0.0001) and CHP (GT: 19.7% *versus* 43.2%, p = 0.0001; TT: 0.7% *versus* 15.9%, p < 0.0001; T: 10.6% *versus* 37.5% p < 0.0001 (Supplementary Table 1).

## Discussion

Pathogenic similarities amongst diseases linked to ILD have been identified^5,27^. In this regard, an excess of mutations in genes previously associated with IPF has recently been detected in RA-ILD^28^, raising the question of whether a common genetic background underlies ILD related entities.

Taking into account these considerations, we aimed to evaluate for the first time the potential implication of *MUC5B* rs35705950 polymorphism, associated with IPF^4^, RA-ILD^6^ and CHP^7^, in the pathogenesis of ASSD, a disease characterised by a high ILD incidence. Our results showed no influence of this genetic variant in the susceptibility to ASSD. Furthermore, no specific association of *MUC5B* rs35705950 with clinical features of ASSD was observed in our study, indicating that this polymorphism does not represent a risk factor for the severity of the disease, and especially for ASSD-ILD+ patients. Additionally, no significant differences in *MUC5B* rs35705950 were also observed in ASSD-ILD+ patients with an UIP pattern when compared to those with a non-UIP pattern. Interestingly, statistically significant differences in *MUC5B* rs35705950 frequencies between ASSD-ILD+ patients and those with ILD unrelated to ASSD (particularly IPF, RA-ILD and CHP) were found. This data suggests a different genetic predisposition between these conditions.

In keeping with our results in ASSD and unlike RA-ILD, no association of *MUC5B* rs35705950 with ILD in the setting of other connective tissue diseases was found. With respect to this, no influence of this genetic variant on myositis-ILD was disclosed whereas genetic differences regarding *MUC5B* rs35705950 between myositis-ILD and idiopathic ILD were also reported^29^. Likewise, lack of association between this genetic variant and ILD in the context of sarcoidosis^30^ and systemic sclerosis^30,31^ was described while differences in *MUC5B* rs35705950 frequencies were found when patients with sarcoidosis-ILD^30^ and those with systemic sclerosis-ILD^30,31^ were compared to patients with IPF.

Despite parallelisms amongst the different phenotypes of ILD, many clinical and radiological differences have been established^5,27,32^. In this regard, a NSIP pattern is more commonly found in ASSD and most connective tissue diseases-related ILD^5,33^, whereas a pattern of UIP is more frequently observed in patients with IPF and RA-ILD^28^. Because of that, it is possible that *MUC5B* rs35705950 may modulate phenotype differences amongst these conditions, suggesting that this genetic variant is not related to shared fibrotic mechanisms across diseases related to ILD, but is instead associated with an IPF and RA-ILD specific pathway. A potential bias may exist in our study regarding the fact that declarative data on the European ancestry were collected from patients and controls included.

In summary, our study does not support a role of *MUC5B* rs35705950 in ASSD pathogenesis. It also indicates that there are genetic differences between ILD associated with and that unrelated to ASSD.

## Supplementary information

Supplementary Information.
