# Supplementary material for: Influence of MUC5B gene on antisynthetase syndrome
Source: Sci Rep. 2020 Jan 29;10:1415. doi: 10.1038/s41598-020-58400-0 (PMC6989632; doi:10.1038/s41598-020-58400-0)
Supplement: Supplementary file 1 — Supplementary Information. [file 41598_2020_58400_MOESM1_ESM.pdf]

Influence of *MUC5B* gene on antisynthetase syndrome

Raquel López-Mejías[1*¥], Sara Remuzgo-Martínez[1¥], Fernanda Genre[1¥], Verónica Pulito-Cueto[1], Sonia M. Fernández Rozas[1], Javier Llorca[2], David Iturbe Fernández[1], Víctor M. Mora Cuesta[1], Norberto Ortego-Centeno[3], Nair Pérez Gómez[4], Antonio Mera-Varela[4], Julia Martínez-Barrio[5], Francisco Javier López-Longo[5], Verónica Mijares[1], Leticia Lera-Gómez[1], María Piedad Usetti[6], Rosalía Laporta[6], Virginia Pérez[7], Alicia De Pablo Gafas[7], María Aránzazu Alfranca González[8], Jaime Calvo-Alén[9], Fredeswinda Romero-Bueno[10], Olga Sanchez-Pernaute[10], Laura Nuño[11], Gema Bonilla[11], Alejandro Balsa[11], Fernanda Hernández-González[12], Ignacio Grafia[13], Sergio Prieto-González[13], Javier Narvaez[14], Ernesto Trallero-Araguas[15], Albert Selva-O'Callaghan[15], Oreste Gualillo[16], Santos Castañeda[17], Lorenzo Cavagna[18], José M. Cifrian[1§], Miguel A. González-Gay[1,19,20§]

Supplementary Table 1. Genotype and allele frequencies of *MUC5B* rs35705950 in ASSD-ILD+ patients and patients with ILD unrelated to ASSD.

MUC5B rs35705950	Genotypes			Alleles		Allele test	
	GG	GT	TT	G	T	p	OR [95% CI][a]
ASSD-ILD+, % (n)	79.6 (113)	19.7 (28)	0.7 (1)	89.4 (254)	10.6 (30)	**<0.0001**	0.21 [0.13-0.33]
Whole cohort of ILD unrelated to ASSD, % (n)	37.5 (87)	53.4 (124)	9.1 (21)	64.2 (298)	35.8 (166)		
ASSD-ILD+, % (n)	79.6 (113)	19.7 (28)	0.7 (1)	89.4 (254)	10.6 (30)	**<0.0001**	0.21 [0.13-0.33]
IPF, % (n)	34.4 (56)	58.8 (94)	6.9 (11)	63.9 (206)	36.0 (116)		
ASSD-ILD+, % (n)	79.6 (113)	19.7 (28)	0.7 (1)	89.4 (254)	10.6 (30)	**<0.0001**	0.26 [0.12-0.55]
RA-ILD, % (n)	48.1 (13)	40.7 (11)	11.1 (3)	68.5 (37)	31.5 (17)		
ASSD-ILD+, % (n)	79.6 (113)	19.7 (28)	0.7 (1)	89.4 (254)	10.6 (30)	**<0.0001**	0.20 [0.11-0.36]
CHP, % (n)	40.9 (18)	43.2 (19)	15.9 (7)	62.5 (55)	37.5 (33)		

ASSD: antisynthetase syndrome; ILD: interstitial lung disease; OR: odds ratio; CI: confidence interval; IPF: idiopathic pulmonary fibrosis; RA: rheumatoid arthritis; CHP: chronic hypersensitivity pneumonitis.

Statistically significant results are highlighted in **bold.**

[a]For the minor allele.
